# Comparison of [18F] NaF PET/CT dynamic analysis methods and a static analysis method including derivation of a semi-population input function for site-specific measurements of bone formation in a population with chronic kidney disease-mineral and bone disorder

**DOI:** 10.1186/s13550-021-00859-7

**Published:** 2021-11-22

**Authors:** M. H. Vrist, J. N. Bech, T. G. Lauridsen, C. A. Fynbo, J. Theil

**Affiliations:** 1 University Clinic in Nephrology and Hypertension, Gødstrup Hospital, Herning, Denmark; 2 Aarhus University, Aarhus, Denmark; 3 Department of Nephology, Aalborg University Hospital, Aalborg, Denmark; 4 Department of Nuclear Medicine, Gødstrup Hospital, Herning, Denmark; 5 Department of Clinical Medicine, Aarhus University, Aarhus, Denmark

**Keywords:** NaF, PET/CT, Hawkins, Patlak, Semi-population input function, CKD-MBD

## Abstract

**Purpose:**

The purpose of this study is to compare dynamic and static whole-body (WB) [^18^F]NaF PET/CT scan methods used for analysis of bone plasma clearance in patients with chronic kidney disease-mineral and bone disorder (CKD-MBD).

**Methods:**

Seventeen patients with CKD-MBD underwent a 60-min dynamic scan followed by a 30-min static WB scan. Tracer kinetics in four thoracic vertebrae were analysed using nonlinear regression and Patlak analysis using image-derived arterial input functions. The static WB scan was analysed using a simplified Patlak method requiring only a single data point in combination with a fixed y-intercept value (*V*_0_), both obtained using a semi-population function. The semi-population function was constructed by combining a previously derived population input function in combination with data from venous blood samples. Static WB scan analysis data, obtained from the semi-population input functions, was compared with paired data obtained using dynamic input functions.

**Results:**

Bone plasma clearance (*K*_i_) from Patlak analyses correlated well with nonlinear regression analysis, but *K*_i_ results using Patlak analysis were lower than *K*_i_ results using nonlinear regression analysis. However, no significant difference was found between *K*_i_ obtained by static WB scans and *K*_i_ obtained by dynamic scans using nonlinear regression analysis (*p* = 0.29).

**Conclusion:**

Bone plasma clearance measured from static WB scans correlates with clearance data measured by dynamic analysis. Static [18F]NaF PET/CT scans can be applied in future studies to measure K_i_ in patients with CKD-MBD, but the results should not be compared uncritically with results obtained by dynamic scan analysis.

## Background

Fluorine-18 labelled sodium fluoride (^18^F-NaF) was introduced in the 1960s as a bone-seeking tracer in nuclear medicine [1] and has been widely used for gamma camera bone scintigraphy. It was replaced by Technetium-99 m labelled diphosphonates in the 1970s. In the past decades, ^18^F-NaF has been reintroduced in combination with PET/CT.

Chronic kidney disease (CKD) is associated with universal bone abnormalities (renal osteodystrophy) and disturbances in mineral metabolism leading to cardiovascular and extra-skeletal calcifications. These processes are caused by common pathophysiological pathways and are defined as a systemic disorder named chronic kidney disease-mineral and bone disorder (CKD-MBD) [2]. Today, bone biopsy is considered the gold standard for analysis [3], but it is invasive, unpleasant and not without risk to the patient. And even though bone-related complications have been known in CKD since 1883 [4], the key to effective treatment and prevention remains to be found. This shortcoming can be possibly attributed to the lack of non-invasive methods available for research in this field, as recruitment to, for example, new treatment monitoring studies which include bone biopsy, is at best difficult.

However, since the 1990s, non-invasive ^18^F-NaF PET/CT has shown good potential for monitoring CKD-MBD [5]. Here ^18^F-NaF, administered as an intravenous bolus injection, diffuses from the plasma into the extravascular compartment in bones from where it is further incorporated into the hydroxyapatite skeleton of bone as fluoroapatite [6].

**Fig. 1:**
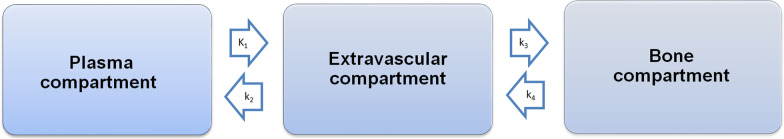
Two-tissue compartment model [7]

The plasma clearance of ^18^F-NaF to bone [*K*_i_ (ml min^−1^ ml^−1^)] predominantly reflects changes in bone formation rate. Traditionally, nonlinear regression analysis with the two-tissue compartment model described by Hawkins (Fig. 1) has been used to determine *K*_i_ and other kinetic parameters using dynamic data acquisition and, as such, is considered the gold standard for dynamic analysis [7, 8]. *K*_i_ values have been shown to correlate well with turnover results obtained by bone biopsies [5]. Dynamic analyses require acquisition over a single field of view (FOV), typically of 60-min duration. Due to restrictions in the axial width (16–25 cm) of almost all but the most modern dynamic whole-body scanners, dynamic scans can examine only a selected region of the skeleton within a single FOV, with extended regions of interest requiring multiple scans and tracer injections. This not only is impractical in a clinical setting but will also result in a high radiation dose to the patient. Previously, this limitation has been addressed through development of a simplified acquisition and analysis method in which a static WB ^18^F-NaF PET scan in combination with a standardized semi-population input function (SPIF) estimates *K*_i_ values in multiple bone regions using a static scan analysis as described previously [9, 10]. The static analysis method is a simplified Patlak analysis with only one data point combined with a fixed intercept value, the fixed value being found from reanalysis of the dynamic scans with the Patlak multiple data point graphical method [8]. Using the static method, it is possible to perform a 30-min WB acquisition with site-specific bone analysis using a single tracer injection [9, 11, 12]. In the clinical setting, this is preferable as firstly, the relatively short scan duration is advantageous with often unwell CKD-MBD patients and secondly, all clinically relevant locations can be studied using only a single tracer injection with a single scan and additionally, it is not so disruptive to the planning of an efficient, high-throughput clinical daily programme compared to the longer, dynamic scan method.

To our knowledge, a systematic study including derivation of a population residual curve for patients with CKD-MBD has not previously been published. The objective of the present study was to compare WB scan *K*_i_ results with *K*_i_ results from dynamic scan analysis to validate the future use of static scans in the CKD-MBD population. Firstly, we implemented the ^18^F-NaF PET/CT scan using the Hawkins method and Patlak analysis for dynamic studies of bone plasma clearance in our clinic for patients with CKD-MBD. Secondly, we derived a CKD-MBD population residual curve for subsequent SPIF construction in combination with venous blood samples to be used as the arterial input function (AIF) for static scan analysis in WB scans. Finally, the static WB analysis was compared with dynamic nonlinear regression.

## Materials and methods

### Subjects

**Fig. 2:**
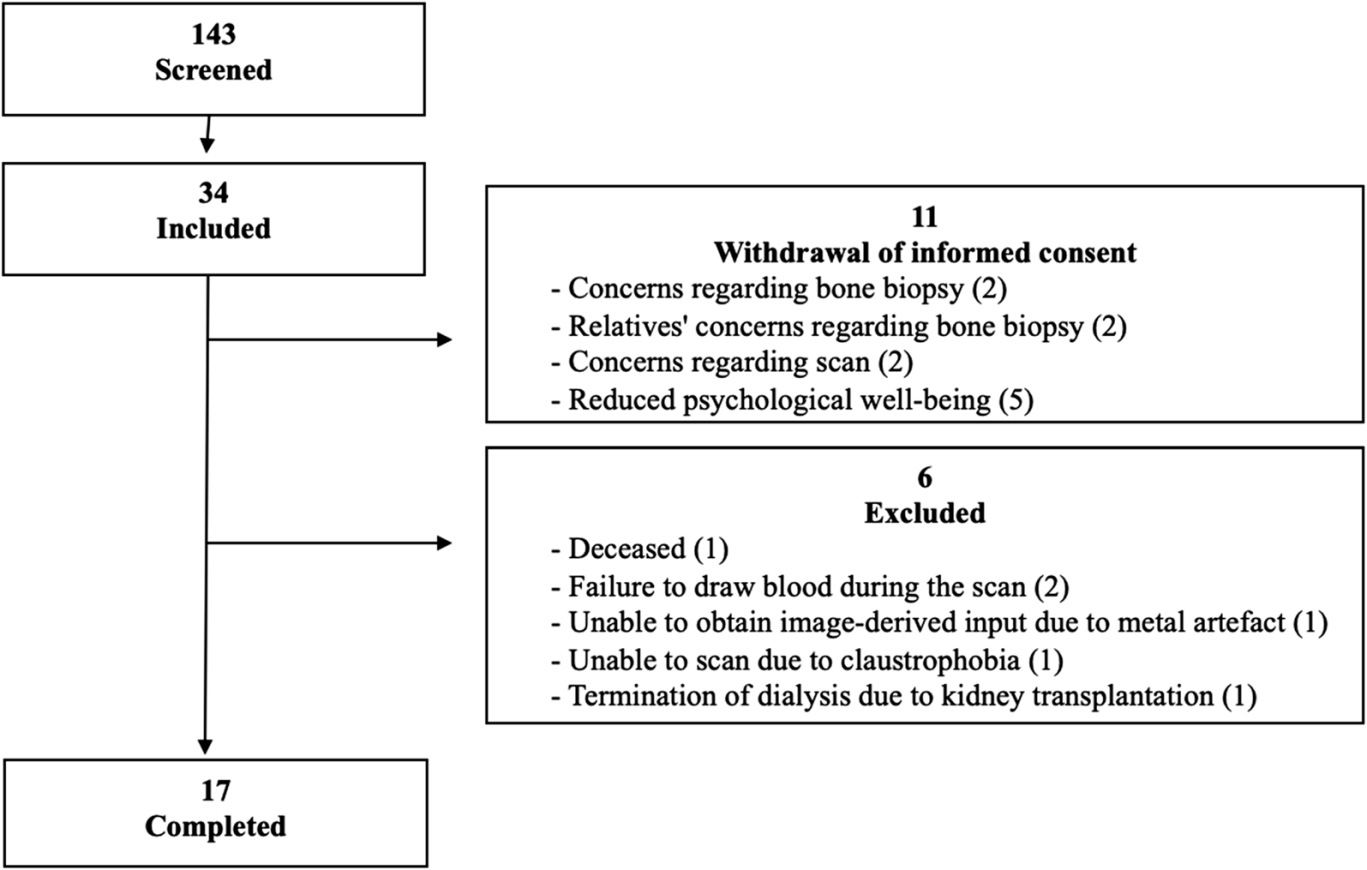
Flow chart of participant recruitment. Patients were also screened to participate in a bone biopsy as part of the complete study. Concerns regarding the bone biopsy contributed largely to the large withdrawal of consent seen in this study

Thirty-four chronic dialysis patients (30–80 years) were included of whom 17 completed the study (haemodialysis *n* = 15, peritoneal dialysis *n* = 2). Exclusion criteria were pregnancy, participants who suffered from alcohol/drug abuse, allergy to ^18^F-NaF or tetracycline, or had suffered bone fracture, acute myocardial infarction, transitory cerebral ischaemia, kidney transplant or parathyroidectomy in the course of the past three months. Table 1 shows demographics, and Fig. 2 shows the flow chart for inclusion.

**Table 1:** Demographics

Demographics (*n* = 17)	
Age, years	62.5 ± 10.1
Female, % (*n*)	29.4 (5)
Male, % (*n*)	70.6 (12)
Body mass index (BMI), kg/m^2^	24 (23–29)
Dialysis duration, years	2.0 (0.5–3.0)
*Cause of kidney failure, % (n)*	
Hypertension	23.5 (4)
Diabetic nephropathy	29.4 (5)
Polycystic kidney disease	23.5 (4)
Glomerulonephritis	5.9 (1)
Other/unknown	17.6 (3)

Normally distributed data: mean ± SD. Non-normally distributed data: median (25th percentile; 75th percentile)

During data analysis, one patient had to be excluded due to very poor image quality resulting from severe obesity and three patients were excluded due to delayed bolus injection or problems with blood sampling.

### Image acquisition

PET/CT images were acquired on a Siemens Biograph mCT-4R 64 slice PET/CT scanner with a 22 cm axial FOV. The participants were positioned with the heart and the thoracic vertebrae 7 through 10 (Th7–Th10) centred in the FOV.

Following an intravenous bolus injection of 150 MBq ^18^F-NaF flushed with 20 mL isotonic saline, a 60-min list-mode dynamic scan was acquired followed by a WB scan from the middle of the femur to the vertex of the skull acquired in 6–7 FOVs of 3 min per bed position.

### Image reconstruction

PET images for dynamic analysis were re-binned into 50 time frames: 20 × 3 s, 12 × 5 s, 4 × 30 s and 14 × 240 s. Reconstruction of PET scans was done using filtered back-projection with a Gaussian filter of 5 mm and a matrix size of 256 × 256.

Low-dose CT scans were performed, and the images were reconstructed in three utilization-dependent series: (1) attenuation correction, (2) localization and identification of thoracic vertebrae in the dynamic scan and (3) localization of the relevant bone regions in the WB scan.

All dynamic images were automatically decay corrected to the study injection time (study reference time). Image data from the WB scan were automatically decay corrected to the start of the WB scan, on average 66 ± 2-min postinjection (mpi), requiring additional decay correction to the study reference time for comparison with dynamic data.

### Blood samples

**Fig. 3:**
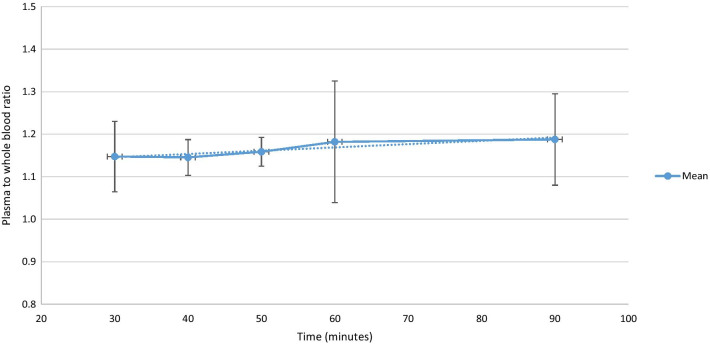
Average plasma-to-whole blood ratio (± 1 standard deviation) over time after tracer injection (*n* = 13)

Venous blood samples (5 mL) were collected at − 5, 30, 40, 50, 60 and 90 min after injection and thereafter centrifuged at 3000 rpm for 10 min. The activity concentrations in 1 ml of whole blood and plasma were measured in a well counter (PerkinElmer Wizard2®-2480 Automatic Gamma Counter, USA). The well counter and PET/CT scanner were cross-calibrated. To convert measured activity from image-derived whole blood to plasma activity curves, plasma-to-whole blood activity ratios were calculated for each of the samples (Fig. 3).

### Image analysis

The PMOD® version 4.003 software (PMOD Technologies LLC, Switzerland) was used for nonlinear regression analysis of the dynamic data and analysis of the static WB data.

**Fig. 4:**
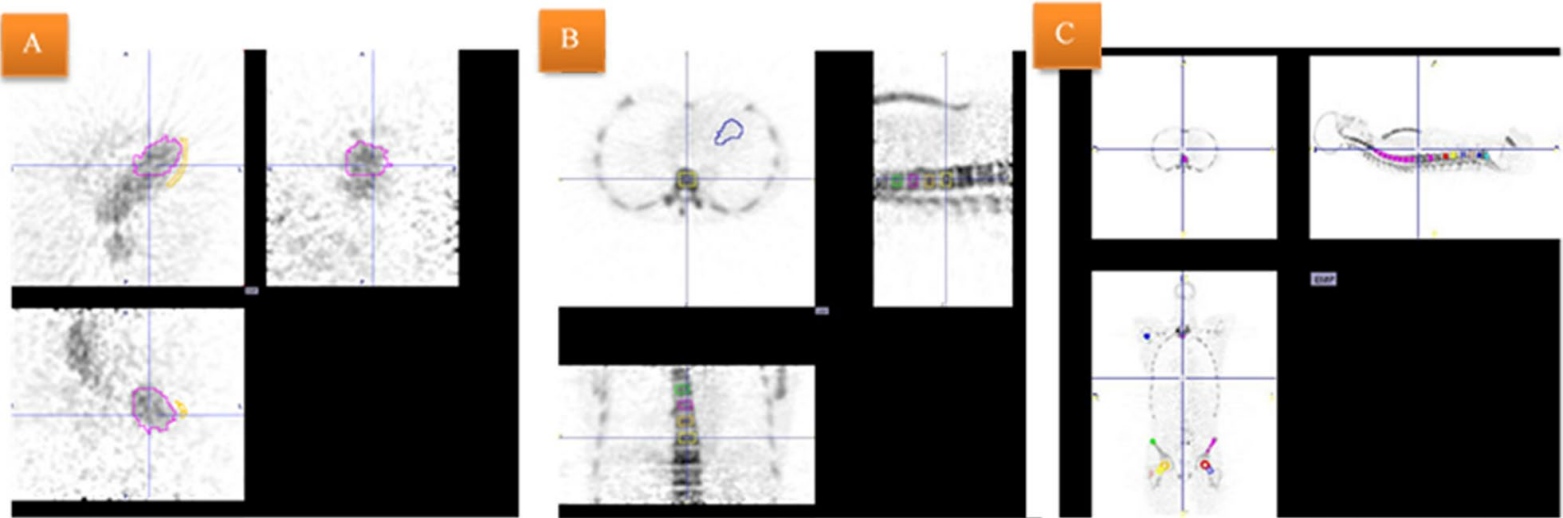
**A** A VOI placed in LV and a myocardial VOI for β-correction. **B** FOV from a dynamic ^18^F-NaF PET/CT showing VOIs to obtain bone TACs in vertebrae Th7–10 and a VOI in the left ventricle of the heart (LV) to obtain the image-derived input function. **C** Whole-body ^18^F-NaF PET/CT with the possibility to make bone VOIs in the entire skeleton

The drawing of VOIs, for generation of time activity curves (TACs) from the cardiac left ventricle (LV), was facilitated by summing the relevant time frames with the highest activity into one static frame. Contours of the activity within the LV were drawn using the hot contouring tool in PMOD, defined as a percentage of the maximum activity within a box VOI surrounding the LV (typically 45–65%) (Fig. 4A). Despite their rather excellent localization quality, our low-dose CT images did not allow for delineation of the ventricular cavity or the myocardial wall as the contrast between the blood-filled cavities and solid walls was non-existent.

For correction of partial volume effect (PVE) and activity spillover/spill-in, VOI contours within the myocardial wall were manually drawn using the brush tool on at least 10 axial slices with a voxel size of 3.2 × 3.2 × 1.4 mm.

Box VOIs were fitted to the trabecular part of the vertebral bodies Th7-Th10, avoiding the endplates and the disc spaces using the CT images as a template. These VOIs were similar for both dynamic and WB studies (Fig. 4B, C).

TACs were derived in the PMOD View Module and transferred to the PMOD Kinetic Module for further analysis as described in detail below.

### Input functions for dynamic scan analysis

Dynamic scan analysis used an image-derived arterial input function (IDAIF) from a VOI placed in the LV as described above. In PMOD, we used a 3-exponential model with fitting of the curve data from peak time and onwards.

**Fig. 5:**
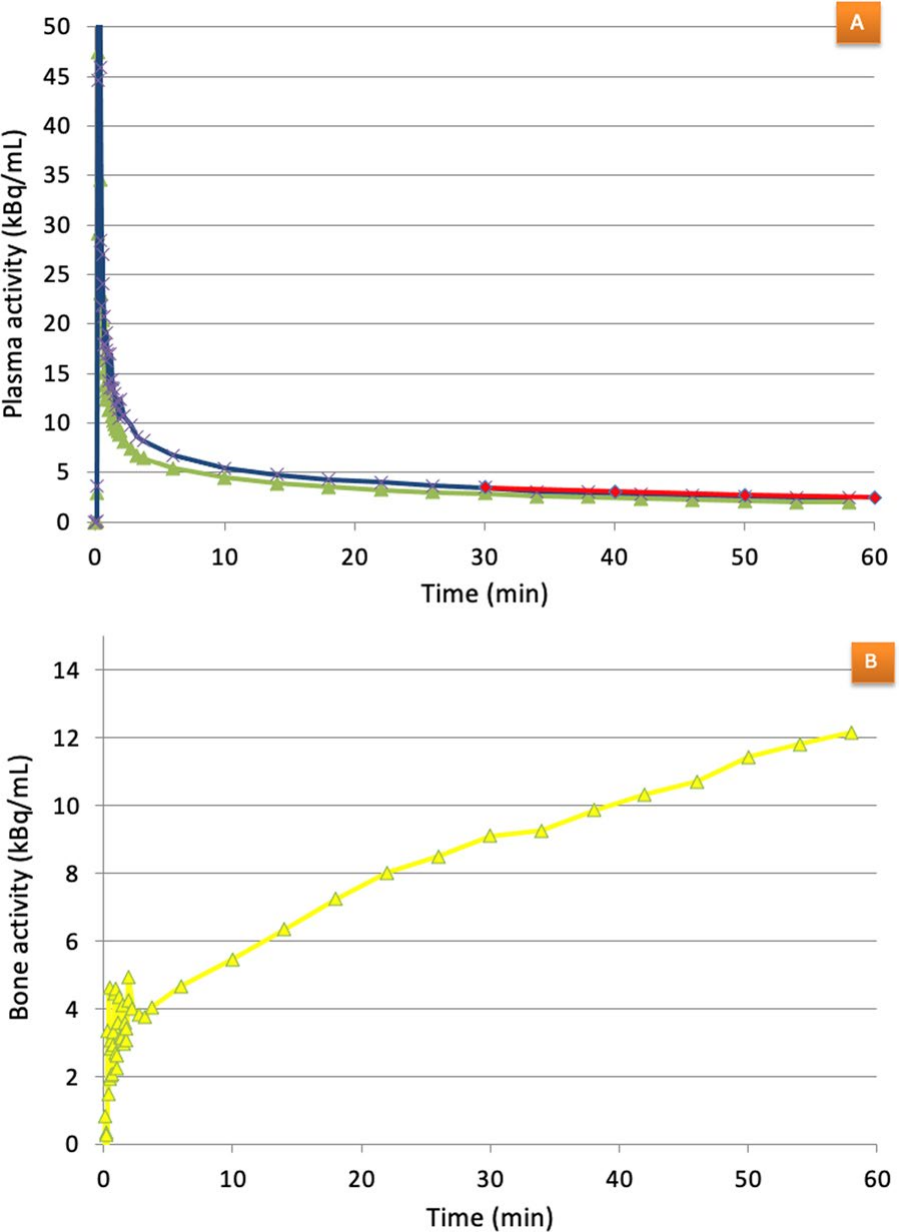
Example data curves obtained in the present study. **A** Green line: non-β-corrected IDAIF obtained from left ventricle. Red line: The exponential obtained from venous blood samples at 30, 40, 50 and 60 min. Blue line: β-corrected IDAIF obtained from left ventricle. **B** Bone TAC obtained from a VOI in thoracic vertebra 9. Noise is present in the initial part of the curve

Errors due to counting efficiency, PVE and spillover/spill-in of activity were corrected by calculation of the recovery coefficient *β*, as described by Cook et al. and Puri et al. [10, 13]:

1 $$R_{{{\text{LV}}}} \left( t \right) = \beta \cdot C_{{{\text{LV}}}} \left( t \right) + \left( {1 - \beta } \right) \cdot C_{{{\text{Bg}}}} \left( t \right)$$

where R_LV_ is activity in the image-derived TAC obtained from the LV, *C*_Bg_ is the background activity concentration in the surrounding myocardium and C_LV_ is the true activity concentration in the LV. *C*_LV_ is approximated to activity in venous blood samples 30 mpi [10]. From (Eq. 1), *β* can be estimated as the mean value of *β*-coefficients calculated from each of the paired image and blood sample data (Fig. 5A).

### Input functions for static scan analysis

For analysis of the static WB scans, we used a 3-exponential SPIF as previously described by Blake et al. [9].

The SPIF was a combination of a population residual curve scaled for the patient-injected activity and added to the terminal exponential derived from the plasma samples. We defined both the 0–60-min SPIF for comparison with the dynamic IDAIFs and the 0–90-min SPIF for use as input functions with static scan studies following the dynamic studies.

The population residual curve was derived from the IDAIFs scaled to a reference dose of 150 MBq for each participant. The residual curve represents the sum of the early fast exponentials and was derived by subtracting the terminal exponential (all data values ≥ 30 mpi) from the entire image-derived curve. All residual curves were adjusted so that the times of peak count rate for all curves were coincident with the most frequent unadjusted peak time (16.5 s). The residual curves were then averaged to define the population residual curve.

### Bone plasma clearance

The bone plasma clearance [*K*_i_ (ml min^−1^ ml^−1^)] was calculated as the mean value of four thoracic vertebrae (Th7–Th10) for the systematic application of three different analytic methods as described below. No correction for delay was made as we found the delay to be of only a few seconds and attempts at correcting the very noisy data in the first few acquisition frames failed to make the data more consistent.

#### Nonlinear regression analysis

PMOD software was used to perform two-tissue compartment dynamic analysis of NaF turnover as described by Hawkins et al. [7]. The exchange of ^18^F-NaF between the compartments: plasma, extravascular and bone (Fig. 1) is described by the kinetic parameters *K*_1_–*k*_4_ and the parameter for regional bone plasma clearance *K*_i_ is defined as:

2 $$K_{i} = \frac{{K_{1 } k_{3} }}{{k_{2} + k_{3} }}.$$

The fractional blood volume *V*_B_ of the bone was fixed at 5% [5].

#### Patlak analysis

Assuming the efflux rate constant *k*_4_ to be negligibly small (*k*_4_ ≈ 0 min^−1^), the Patlak graphical analysis provides a simpler alternative analysis method for measuring *K*_i_ as described by Eq. 3 [8, 14]:

3 $$\frac{{C_{{{\text{Bone}}}} \left( T \right)}}{{C_{{{\text{Plasma}}}} \left( T \right)}} = K_{i} \frac{{\mathop \smallint \nolimits_{0}^{T} C_{{{\text{Plasma}}}} \left( t \right){\text{d}}t}}{{C_{{{\text{Plasma}}}} \left( T \right)}} + V_{0} .$$

This equation approximates a straight-line fit with *K*_i_ as the slope. *C*_Bone_ and *C*_Plasma_ are the respective concentrations of tracer bound in bone and freely diffusible in plasma at each time point (*t*). *V*_0_ is the intercept of the ordinate and represents the apparent volume of distribution.

*K*_i_ was calculated from the 60-min dynamic PET/CT scan using a bone TAC and IDAIFs or SPIFs.

#### Static scan analysis

The static scan analysis requires a single data point using activity measured in a selected bone region combined with a previously determined value of the intercept (*V*_0_) for calculation of the straight-line function with *K*_i_ as the slope [11].

### Statistical analysis

Normally distributed results are presented as mean ± standard deviation (SD). Non-normally distributed data are presented as median (25th percentile; 75th percentile).

The paired *t*-test was used to compare means, where a two-tailed *p* value of 0.05 or less was considered statistically significant. Correlations between *K*_i_ values obtained using different analysis models were calculated by linear regression and presented by Pearson’s correlation coefficient, and the Chi-squared test was used to evaluate the fit of the input functions to an applied model. The percentage coefficient of variation of the population residual curves was obtained by dividing the SD by the population residual curve, and the 95% confidence interval was estimated using a Chi-squared distribution.

## Results

### Input functions

The mean plasma-to-whole blood ratio was 1.17 ± 0.03. As shown in Fig. 3 the ratio was variable with only a slight increase from 30 to 90 min (slope 0.0008, intercept 1.12).

The mean recovery coefficient β was 0.69 ± 0.15 when the IDAIF was obtained from TACs in the LV. Table 2 evaluates the IDAIF by comparing the terminal exponentials with the terminal exponential of the plasma samples, and we found no differences in activity values at all comparable time points to the 30–60-min blood samples. The alignment between the terminal exponential from input functions derived by image and by blood samples is pictured in Fig. 5A.

**Table 2:** Comparison of the terminal exponentials of blood samples and the terminal exponential of image-derived input curve

		Blood sample-derived terminal exponential
		30, 40, 50 and 60 min
Image-derived terminal exponential	Mean difference (kBq/min) ± SD	− 0.32 ± 0.79
	*p* value	0.187
	Mean ratio ± SD	1.06 ± 0.14
	*p *value	0.143

Mean differences significantly different from 0 and mean ratios significantly different from 1 were determined using the student paired *t*-test. Level of statistical significance *p* < 0.05

**Fig. 6:**
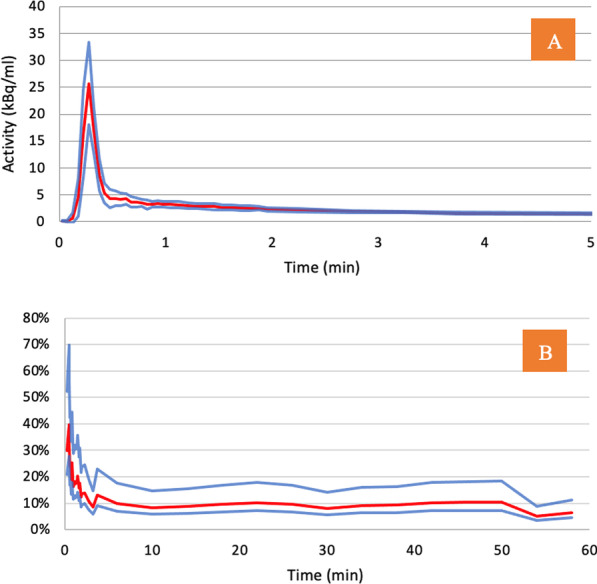
**A** Population residual curve (red line) ± 1SD (blue lines). All curves are normalized to a reference activity of 150 MBq. **B** The corresponding population SDs (red lines) plotted as the percentage coefficient of variation obtained by dividing the SD by the population residual curve with 95% confidence intervals (blue lines) estimated using the Chi-squared distribution

The derived population residual curve is shown in Fig. 6A. The variation in the curve is illustrated by the SD of the residual curves in Fig. 6B (plotted as the percentage of the total population residual curve). The highest SD was at the peak time of the population residual curve with a SD of 30% (95% CI 21–52%) and an average SD of the entire curve of 15.8%.

Table 3 shows the characteristic areas under the curve (AUC) values of the various image-derived AIFs and SPIFs. No difference was found between AUCs comparing IDAIF and SPIFs (*p* values: 0.67 and 0.315). Additionally, no difference was found between AUCs of IDAIFs corrected with 30–60-min blood samples (353 ± 59) compared with AUCs of IDAIFs corrected with 30–90-min blood samples (341 ± 54) (*p* value: 0.09).

**Table 3:** Comparison input functions obtained by β-corrected image-derived arterial input functions (IDAIF) and semi-population input functions

	IDAIF β-corrected and blood samples (30–60 min)	SPIF Population residual and blood samples (30–60 min)	IDAIF β-corrected and blood samples (30–90 min)	SPIF Population residual and blood samples (30–90 min)
AUC ± SD	353 ± 59	348 ± 74	341 ± 54	331 ± 70
Percentage difference (*p* value)		− 1.4% (0.671)		− 2.9% (0.315)

The data are presented as mean values ± SD obtained by the same 12 scans. *AUC* area under the curve. Level of statistical significance is *p* < 0.05

### Bone plasma clearance

Figure 5B shows a typical bone TAC. Initially, the curve is poorly defined as frame counts are low. Uptake in bone rises significantly after a few minutes, but with a slowly declining rate towards the end of the examination.

#### Nonlinear regression analysis

The rate constants (*K*_1_–*k*_4_) determined using the two-tissue compartment model are shown in Table 4. The mean value of *k*_4_ was 0.005 min^−1^ which indicates that—at least within the 60-min time frame of the dynamic study—the efflux of ^18^F-NaF from the vertebrae is low, an important prerequisite for proper use of the Patlak analysis method.

**Table 4:** Results of nonlinear regression analysis for the rate constants (K_1-4_) using the two-tissue compartment model

*K*_1_ (ml min^−1^ ml^−1^)	*k*_2_ (min^−1^)	*k*_3_ (min^−1^)	*k*_4_ (min^−1^)	Chi^2^
0.111 ± 0.04	0.366 ± 0.26	0.195 ± 0.07	0.005 ± 0.005	60.1 ± 53.4

Goodness of fit for the curves was evaluated by Chi-squared test. Values from 12 scans are expressed as mean ± SD. Image-derived AIFs (IDAIFs) were obtained from a TAC of left ventricle of the heart (LV)

#### Patlak analysis

**Fig. 7:**
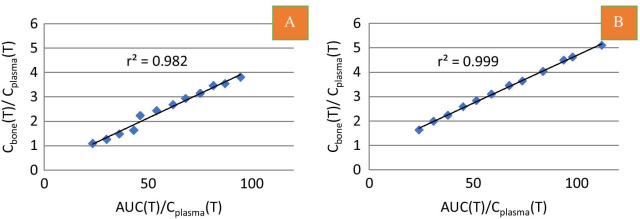
Correlation of dynamic Patlak coordinates using IDAIF. **A** Lowest correlation coefficient found at participant no 2, vertebrae th9. **B** Highest correlation coefficient found at participant no. 21, vertebra Th10

All dynamic datasets analysed according to Eq. 3 showed excellent straight-line fits with linear correlation coefficients close to 1.0 (average *R*^2^ = 0.996 ± 0.002) (Fig. 7).

Table 5 compares *K*_i_ values obtained from nonlinear regression analysis and Patlak analysis using both IDAIFs and SPIFs. *K*_i_ values were lower using the Patlak analysis than *K*_i_ values obtained from nonlinear regression analysis. No difference was found in *K*_i_ values when IDAIFs or SPIFs were applied for Patlak analysis.

**Table 5:** Comparison of K_i_ values obtained by nonlinear regression analysis with Ki values obtained by Patlak analysis using various input functions or static scan analysis

	Nonlinear regression	Patlak analysis			Static scan
	IDAIF	IDAIF	SPIF (blood samples 30–60 min)	SPIF (blood samples 30–90 min)	SPIF (blood samples 30–90 min)
*K*_i_ (ml min^−1^ ml^−1^) ± SD	0.0415 ± 0.013	0.0337 ± 0.009	0.0340 ± 0.008	0.0346 ± 0.009	0.0395 ± 0.011
Percentage difference (*p*-value)	–	− 18.8% (< 0.01*)	− 18.1% (< 0.01*)	− 16.6% (< 0.01*)	− 4.8% (0.29)
V_0_ ± SD	–	0.39 ± 0.17	0.52 ± 0.25	0.64 ± 0.27	0.64

Mean ± SD from 12 scans (Th7–10). *Level of statistical significance is *p* < 0.05. Image-derived AIFs (IDAIFs) were obtained from a TAC of left ventricle of the heart

**Fig. 8:**
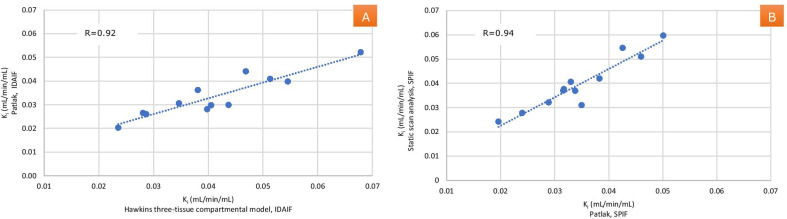
**A** Correlation between mean *K*_i_ values obtained by nonlinear regression analysis and Patlak analysis. **B** Correlation between mean *K*_i_ value obtained by Patlak analysis of dynamic scan and mean *K*_i_ value obtained by static scan

Comparison of *K*_i_ results between nonlinear regression analysis and Patlak analysis using *β*-corrected IDAIF in combination with 30–90-min blood samples (Fig. 8A) shows a statistically significant linear correlation (*R*^2^ = 0.92, *p* < 0.001). Similarly, a statistically significant correlation was found between nonlinear regression analysis and Patlak analysis *K*_i_ values using β-corrected IDAIF plus 30–60-min blood samples (*R*^2^ = 0.93, *p* < 0.001).

#### Static scan analysis

Table 5 shows no difference between *K*_i_ values obtained by static scan analysis and *K*_i_ values obtained by dynamic nonlinear regression. Table 6 shows that *K*_i_ values analysed by static scan analysis were higher than dynamic *K*_i_ values analysed by Patlak analysis, but with a good correlation.

**Table 6:** *K*_i_ results obtained from static scan using the static scan analysis compared with *K*_i_ results obtained from dynamic scan using Patlak analysis

Scan	First and second exponentials	Terminal exponential	V_0_ (%)	K_i_ (ml min^−1^ ml^−1^)	Percentage difference	*p* Value	*R*	*p* value
Dynamic, Patlak	Population residual	Blood samples 30–90 min	64	0.0346 ± 0.009	–	–	–	–
Static	Population residual	Blood samples 30–60 min	43	0.0399 ± 0.010	+ 15%	< 0.001*	0.930	< 0.001*
			52	0.0390 ± 0.010	+ 13%	0.002*	0.931	< 0.001*
Static	Population residual	Blood samples 30–90 min	43	0.0419 ± 0.011	+ 21%	< 0.001*	0.940	< 0.001*
			64	0.0395 ± 0.011	+ 14%	0.001*	0.942	< 0.001*

Mean values ± SD are presented from 12 scans (Th7–10). Three different assumptions of V0 are applied and compared with Patlak analysis Ki values obtained using dynamic scans. Pearson’s correlation coefficient (R), *Level of statistical significance: *p* < 0.05

Figure 8B shows a statistically significant correlation between dynamic Patlak analysis and static scan analysis of mean *K*_i_ values using SPIF from 30 to 90-min blood samples (*R* = 0.94, *p* < 0.001). A significant correlation was also observed when using 30–60-min blood samples (*R* = 0.87, *p* < 0.001).

## Discussion

Methods for assessing skeletal health that can replace the gold standard bone biopsy are sorely needed in the clinical CKD-MBD setting; for example, effective bone treatment may be withheld if a patient is suspected of having adynamic bone disease. Access to non-invasive diagnostic methods to confirm or negate this would be an important clinical gain. The present study implements both dynamic and static ^18^F-NaF PET/CT imaging using various analysis methods: nonlinear regression, Patlak analysis, static graphical analysis and construction of a representative CKD-MBD SPIF.

All presented methods are found suitable for bone plasma clearance evaluation; still, the simplified static graphical analysis was found to be preferable for implementation in clinical practice due to its short time scan and its ability to examine multiple bone regions using a single tracer injection, compared to the limitation of a single FOV with dynamic acquisition. We found no difference in K_i_ results obtained by dynamic nonlinear regression or static scan analysis. Static WB ^18^F-NaF PET/CT allows a relatively easy clinical assessment of skeletal variables in CKD-MBD.

To some extent, CKD-MBD is present in all patients suffering from severe kidney failure in need of dialysis treatment. Therefore, the present study includes chronic dialysis patients as representatives of the CKD-MBD population. The participants were mostly males (70%), and diabetic nephropathy was the most common cause of kidney failure (29%). Previous studies using ^18^F-NaF PET/CT for dynamic bone examination have been mostly carried out on female osteoporotic patients and excluded patients with CKD [12, 15]. However, a recently published study of a CKD-MBD population also included 50% males with diabetic nephropathy as the most common cause of kidney failure (34%) [16].

### Input functions for dynamic scan analysis

In the present study, we evaluate different input functions. The mean AUC of a β-corrected IDAIF has previously been shown to be comparable with the AUC from direct arterial sampling [13], and as such, the β-corrected IDAIF was chosen as our reference input function for comparison with results from other input functions. All IDAIFs were converted to plasma activity using the average plasma-to-whole blood ratio found by venous blood sampling as described by Cook et al. [10]. In this present study, we find the plasma/whole blood ratio to be slightly lower. Some of the difference may be explained by renal anaemia in the CKD-MBD population. As we did not have blood samples in the time period of 0–30 min, we were unable to correct for changes in the plasma/whole blood ratio over time as done by Cook et al. [10]. Additionally we found no time dependence of the ratio over the considered time window, as well as a low inter-subject variability, indicating that a single value of 1.17 may be used for data correction in future scans.

The recovery coefficient *β* was calculated to correct the IDAIF for the combined effects of counting efficiency, PVE and spillover/spill-in of activity between activity in the lumen and background structures. We found the variation of this *β*-coefficient to be dependent on image quality and blood sampling errors. Hence, optimal blood sampling is particularly important to obtain a reliable *β*-correction. The present study found a much lower *β*-coefficient (0.69 ± 0.15) than previously published mean *β*-coefficient values (0.97 ± 0.54) in which the aorta was used for derivation of input functions [13]. The reason for this is most probably our use of a large VOI (~ 20 cm^3^) to define the LV input function with a representative sample of the inhomogeneous activity, as a VOI with borders close to the myocardial wall (3–6 mm separation) is subject to PVE and spillover of activity in combination with myocardial movement (5–8 mm motion) as reported by Cho et al., thus exposing it to activity in the myocardial wall [17]. In future studies, this should be remedied by use of a smaller VOI placed in the middle of the LV, which is less subject to PVE and spillover but, on the other hand, more dependent on correct placement around the highest voxel in the inhomogeneous distributed activity. In spite of this, as long as the (local) quantitatively correct *β*-correction value is used for the applied analysis protocol and imaging scanner, the *β*-correction method is shown to define the AUC of the IDAIF as well as with direct arterial sampling [15]. This is to be expected as the level of the terminal exponential of the input function is shown to account for 75% of the AUC of the input function in the first 60 min [9]. Thus, a precise correction of the terminal exponential of the IDAIF is of high interest.

To enable future static scan analysis of multiple bone regions, a SPIF was derived combining the population residual curve and venous blood samples to produce an AIF. No difference was found between AUCs of the SPIFs compared with AUCs of the IDAIFs (Table 3). This suggests that it is feasible for a fully dynamic IDAIF to be replaced by a generalized SPIF for estimation of dynamic information in bone clearance studies, which is necessary for investigation of extended, multiple bone regions (Fig. 4C).

*V*_0_ is observed to vary with the choice of input function as shown in Table 5. Our Patlak analysis yields a mean *V*_0_ value of 0.39 ± 0.17 when IDAIF is applied as input function. This value is comparable to the population value of 0.43 previously reported by Siddique et al. [11] in a population of ten women with osteoporosis. However, when SPIFs are used as input function for Patlak analysis, mean V_0_ values tend to be higher. For static analysis, Siddique et al. have previously published a *V*_0_ of 0.46 when using a SPIF [18]. Thus, our values are in the same range as previously published values.

The value of *V*_0_ is known to be specific to the skeletal site, treatment and analysis model [11, 18, 19]. However, *K*_i_ estimates have been shown to be relatively independent of the choice of *V*_0_. A 20% difference in *V*_0_ resulted in only a 5% change in *K*_i_ [11], making the static analysis robust for clinical use despite variability in the population *V*_0_ value.

### Input functions for static scan analysis

The rationale for using an average population curve calibrated by the individual patient’s plasma activity is the assumption that the shape of each patient's input curves is similar, but the actual plasma values may differ due to, for example, disease or treatment of the disease [12]. The authors show standard uptake value (SUV) analysis is less sensitive to changes in actual bone metabolism as negative changes in arterial activity may almost cancel out positive changes in bone uptake due to treatment in the calculation of SUV, as well as being dependent on factors other than bone uptake (e.g. renal clearance). Previously, two methods have been applied to relate bone uptake to actual plasma activity: either by using the fractional uptake rate (FUR), which is comparable to the standard uptake ratio used in studies of glucose metabolism, or by using a SPIF as in the present study [9, 16, 20]. Furthermore, using a SPIF, instead of a fully obtained IDAIF for each examination, can reduce the effective scan time remarkably.

The standard deviation is generally high in the obtained population residual curve (Fig. 6). The highest SD was at the peak time of the population residual curve with a SD of 30% (95% CI 21–52%) and an average SD of the entire curve of 15.8%. These SDs are comparable with the values of 26.4% (19.0–42.3%) previously published by Blake et al. [9]. However, in the present study, the ^18^F-NaF tracer was administered by manual bolus injection. It may be possible to produce a population residual curve with less variation using an automated injection system.

In the present study, time adjustment to the common peak position of the residual curves to make the average population curve was performed by time shifting the curves so that the frames with maximum activity coincide. This could be suboptimal since the temporal sampling is low and the actual peak position does not coincide with the exact frame position having the maximum activity, potentially resulting in an artificial elevation of the averaged peak. However, the actual peak value of the input function seems to have little importance when calculating *K*_i_, which is instead highly dependent on a reliable input function AUC. The semi-population residual curves and IDAIF show similar AUCs.

Overall, we found the CKD-MBD population residual curve comparable with the previously published population residual curve for osteoporotic patients.

### Bone plasma clearance

The greatest variability in the bone TACs occurs in the initial portion of the curves due to a combination of initial low activity and short time frames (Fig. 5B). Other published studies have used an average TAC over all the vertebra to be investigated with longer time bins for each frame; this improves counting statistics but lowers the time resolution and may, as such, be counterproductive [11, 12]. However, this problem does not affect Patlak analysis, as the data are sampled at later time points between 14 and 60 min.

#### Nonlinear regression analysis

The mean *K*_i_ value was 0.042 ± 0.01 ml min^−1^ ml^−1^ applying IDAIFs in a two-tissue compartment model (Table 5).

The first quantitative ^18^F-NaF study evaluating kinetics in renal osteodystrophy reported a mean *K*_i_ value of 0.071 ± 0.03 ml min^−1^ ml^−1^ [5]. The reason for this high value may be that 72% of the population studied had untreated secondary hyperparathyroidism. Correspondingly, a new study by Aaltonen et al. reported a mean value of 0.067 in dialysis patients with high turnover bone disease and 0.038 in dialysis patients with low turnover bone disease [16].

In comparison, our value of 0.042 ml min^−1^ ml^−1^ lies within the lower cutoff limit defined in the Aaltonen study and above the value reported for two patients with hyperparathyroidism as found by Schiepers et al. (0.034 ml min^−1^ ml^−1^). Additionally, the latest study, which studied *K*_i_ related to Paget disease, published a much higher mean value of 0.114 ml min^−1^ ml^−1^ [21].

#### Patlak analysis

The present study found a statistically significant correlation between the *K*_i_ values obtained using nonlinear regression analysis and Patlak analysis (*R*: 0.92, *p* value: 0.001) (Fig. 8A), as for previously published values in a healthy female population and a postmenopausal female population with osteoporosis [15, 22].

The mean *K*_i_ value was 0.034 ± 0.01 ml min^−1^ ml^−1^ using IDAIFs as input to the Patlak analysis. This is comparable to results published for a chronic dialysis population (*L*_1–4_) with a mean value of 0.039 ml min^−1^ ml^−1^, and as expected, it is higher than the mean value of 0.028 ml min^−1^ ml^−1^ found for a haemodialysis population with suspected adynamic bone disease (*L*_1–4_) [16, 23].

The mean *K*_i_ value was significantly lower when analysed using the Patlak analysis than with nonlinear regression analysis (Table 5). The average paired difference between *K*_i_ values for the two methods is − 17.4 ± 10%. This difference varies and was previously reported to be − 7% by Brenner et al., − 13% by Installé et al. and − 23.7% by Puri et al. [14, 15, 24].

It has been suggested that Patlak analysis results are lower than those derived from the two-tissue compartment model due to efflux of tracer from the bone during the scan. In previous studies, the Patlak plots often showed a small but obvious curvature with the slope decreasing slightly as time advanced, which has been related to tracer efflux. If such efflux is present, it may be corrected by the method described by Siddique et al. [18].

However, in the present study this curvature of the Patlak plot was barely visible and all plots fitted very well to a straight line with regression coefficients close to 1 (Fig. 7). Combined with the small *k*_4_ values of 0.005 with a high standard deviation of ± 0.005, a small tracer efflux can only partly explain the discrepancy between our NLR and Patlak results and we found it disproportionate to correct for such a small efflux in this study.

The Patlak analysis has been reported to be superior to the two-tissue compartment model for research purposes as it is computationally simpler and requires a lower number of participants to show a statistically significant result due to its small precision error [14, 22].

#### Static scan analysis

Using the static scan analysis with a SPIF as described above for the four vertebrae Th7–Th10 imaged in WB scans, we found the *K*_i_ value to be 0.0395 ± 0.011 ml min^−1^ ml^−1^. Thus, no difference was found between the static *K*_i_ results and the dynamic *K*_i_ results obtained by linear regression. Unsurprisingly, the *K*_i_ result in the present study was higher than the *K*_i_ result from a previous published study in patients with suspected adynamic bone disease (0.028 ± 0.012 ml min^−1^ ml^−1^). For comparison, the *K*_i_ result in a study of patients with osteoporosis was in the lower range with a value of 0.025 ± 0.007 ml min^−1^ ml^−1^ [22, 23].

In the present study, comparing *K*_i_ results from the static scan analysis with *K*_i_ results from the Patlak analyses using the same input functions resulted in 14% lower *K*_i_ results (*p* < 0.001) than with the static *K*_i_ results. Despite this, the correlation between the methods is very good (*R*^2^ = 0.942, *p* < 0.001). However, this emphasizes the importance of using the same analysis method when comparing results of tracer kinetic parameters.

The calculation of *K*_i_ in the static scan analysis is dependent on an accurate estimate of the blood/plasma activity at the same time point as which the bone region in question is scanned during the WB acquisition. Neither in the present ^18^F-NaF study, nor in future studies, would we want to exceed a study period of 90 min for the WB scan. However, as we included a venous sample taken immediately after the WB examination (90 < *t* < 100 min, where “*t*” is the time for scanning the bone region in question as obtained from the PMOD analysis tool (see “Methods”), we were able to use a less error prone interpolation, rather than an extrapolation of data, to obtain Cpl(t)/AUC(t) values for calculation of the *K*_i_ value in the study interval between 60 and 90–100 min: We chose to use a standard mono-exponential fit of the terminal data at 40, 50, 60 and 90–100 mpi for interpolation.

However, we could just as well have used a hyperbolic fit of all curve data from 1 mpi and later, providing an even better fit as shown by van den Hoff et al. [25]. This method might be especially important if one is required to extrapolate to data values later than 60 mpi without the benefit of later blood samples.

#### Limitations of the study

The limitations of the present study include not having AIF data from direct arterial sampling as a gold standard to evaluate the validity of the IDAIFs and SPIFs; however, as described above, venous blood samples can complement the arterial sampling curve at least after 30 min [10]. Additionally, the averaging of the residual curves for construction of a population residual curve presented here is not optimal and should be refined in future studies.

In this study design, the static scan sequentially followed a 60-min dynamic scan with the static scan analysis including a 90-min blood sample for construction of an input function ending at time point 90–100 mpi. For this reason, even a small efflux might influence the Patlak plot/static scan analysis presented here, and correction for efflux may be necessary. Thus, in order to minimize effects of possible efflux, our recommendation would be to make the static scan between 30 and 60 min after tracer injection in future studies.

Dynamic scanning in the clinical setting is generally restricted to a single bed position but may change in the future with the advent of new dynamic total body PET scanning combining an initial, short-duration dynamic acquisition over the heart followed by a series of fast, multiple whole-body scans. However, a low, effective scan time ensured by a SPIF and acquisition of a simple static scan may still be a valuable tool with the use of older scanners. Furthermore, a short effective scan time is important in the very sick CKD-MBD population to enable successful completion of an entire WB scan or the static scan time can be reduced to about 5–10 min if only a single bone region, e.g. the lumbar vertebrae, needs to be examined.

## Conclusion

Three different methods for analysis of bone plasma clearance in a population of patients with CKD-MBD for both experimental and clinical use using ^18^F-NaF PET/CT scan have successfully been implemented.

A dynamic scan protocol using the two-tissue compartment model with robust mean *K*_i_- and *K*_1–4_ values has been established, and it was observed that the resulting *K*_i_ values using Patlak analysis were lower than the values obtained from the two-tissue compartment model.

A CKD-MBD population residual curve and corresponding SPIF have been developed for use with WB PET/CT scans, allowing site-specific measurements of bone formation in multiple regions. However, our findings suggest that it is not mandatory for disease-specific population residual curves to be obtained for analysis of *K*_i_.

Of relevance for simple clinical implementation, *K*_i_ values obtained using static WB scanning were found to show no significant difference from *K*_i_ values obtained using nonlinear regression analysis. Thus, a simpler evaluation of bone turnover is now possible in further studies of patients with CKD-MBD; this simpler evaluation may replace the more complicated dynamic scan analysis. Comparison with bone biopsy is required to further validate bone turnover measurements determined from static ^18^F-NaF PET/CT scans in patients with CKD-MBD.
